# Complete Genome Sequences of the Neethling-Like Lumpy Skin Disease Virus Strains Obtained Directly from Three Commercial Live Attenuated Vaccines

**DOI:** 10.1128/genomeA.01255-16

**Published:** 2016-11-10

**Authors:** Elisabeth Mathijs, Frank Vandenbussche, Andy Haegeman, Alasdair King, Bethuel Nthangeni, Christiaan Potgieter, Louis Maartens, Steven Van Borm, Kris De Clercq

**Affiliations:** a Molecular Platform, Veterinary and Agrochemical Research Centre, Ukkel, Belgium; b Viral Diseases, Vesicular and Exotic Diseases, Veterinary and Agrochemical Research Centre, Ukkel, Belgium; c MSD Animal Health, Intergovernmental Veterinary Health, Madison, New Jersey, USA; d Onderstepoort Biological Products, Onderstepoort, South Africa; e Deltamune (Pty) Ltd., Animal Health Products, Product development division, Roodeplaat, South Africa; f Department of Biochemistry, Centre for Human Metabolomics, North-West University, Potchefstroom, South Africa

## Abstract

Lumpy skin disease virus (LSDV) causes an economically important disease in cattle. Here, we report the complete genome sequences of three LSDV strains obtained directly from the live attenuated vaccines: Lumpyvax (MSD Animal Health), Herbivac LS (Deltamune) and Lumpy Skin Disease Vaccine (Onderstepoort Biological Products).

## GENOME ANNOUNCEMENT

Lumpy skin disease (LSD) is an economically important disease in cattle caused by lumpy skin disease virus (LSDV), a member of the *Capripoxvirus* genus. Historically restricted to Africa, the disease has conquered the Middle East and has been making its way into Europe since 2014–2015 (1). Vaccination plays a key role to control the spread of the disease. Here we report the complete genome sequences of three LSDV strains obtained directly from commercial batches of three live attenuated vaccines: Lumpyvax (MSD Animal Health), Herbivac LS (Deltamune), and Lumpy Skin Disease Vaccine (Onderstepoort Biological Products—OBP).

Freeze-dried vaccine pellets were dissolved in 3 mL phosphate-buffered saline (PBS) and DNA was purified using a Puregene Core Kit A (Qiagen) according to the manufacturer’s instructions. Presequencing enrichment was performed through an in-house long-range PCR methodology covering the entire genome with overlapping ~5.5-kb amplicons. P6-C4 sequencing was performed on one single-molecule real-time (SMRT) cell on a PacBio RSII sequencer (Pacific Biosciences) at the Genomics Core UZ Leuven (Belgium).

Consensus amplicon sequences were obtained from the reads using the Long_Amplicon_Analysis protocol (default parameters; Pacific Biosciences) in SMRT Portal version 2.3.0 (Pacific Biosciences). These amplicons were further assembled into unique contigs using iAssembler software (2). Discrepancies with previously published LSDV genomes were confirmed by Sanger sequencing. The protein-coding genes were predicted by NCBI ORF-Finder (http://www.ncbi.nlm.nih.gov/orffinder/) and by GATU relative to the Neethling vaccine LW 1959 sequence (AF409138) (3, 4).

The amplicon sequences of the virus strains from Lumpyvax, Herbivac LS, and Lumpy Skin Disease Vaccine (OBP) assembled into double-stranded, linear DNA contiguous sequences of 150,480 bp, 150,529 bp, and 150,508 bp, respectively. The nucleotide composition of all genomes is 25.91% G+C, evenly distributed. The genomes of all three strains share 99.9% homology with each other but also with the LSDV strain Neethling vaccine LW 1959. All three vaccine strains differ from LW 1959 by two amino acid modifications (T/M in gene LW056 and V/A in gene LW116) and two single-nucleotide deletions that do not affect the coding sequence. Lumpyvax and Herbivac LS both have an additional amino acid difference in gene LW037 (G/V). Additionally, Herbivac LS exhibits two single-nucleotide deletions of which one causes a frameshift that truncates gene LW134a (2,166 instead of 6,075 nucleotides [nt]). LSD vaccine OBP contains an 18-nt deletion in the terminal noncoding region and another three single-nucleotide insertions/deletions that do not affect the coding sequence.

### Accession number(s).

The complete genome sequences of the Neethling-like LSDV vaccine strains from Lumpyvax, Herbivac LS, and LSD vaccine have been deposited in GenBank under accession numbers KX764643, KX764644, and KX764645, respectively.
