# Correction for Mathijs et al., Complete Genome Sequences of the Neethling-Like Lumpy Skin Disease Virus Strains Obtained Directly from Three Commercial Live Attenuated Vaccines

**DOI:** 10.1128/genomeA.01657-16

**Published:** 2017-02-09

**Authors:** Elisabeth Mathijs, Frank Vandenbussche, Andy Haegeman, Alasdair King, Bethuel Nthangeni, Christiaan Potgieter, Louis Maartens, Steven Van Borm, Kris De Clercq

**Affiliations:** a Molecular Platform, Veterinary and Agrochemical Research Centre, Ukkel, Belgium; b Viral Diseases, Vesicular and Exotic Diseases, Veterinary and Agrochemical Research Centre, Ukkel, Belgium; c MSD Animal Health, Intergovernmental Veterinary Health, Madison, New Jersey, USA; d Onderstepoort Biological Products, Onderstepoort, South Africa; e Deltamune (Pty) Ltd., Animal Health Products, Product Development Division, Roodeplaat, South Africa; f Department of Biochemistry, Centre for Human Metabolomics, North-West University, Potchefstroom, South Africa

## AUTHOR CORRECTION

Volume 4, no. 6, e01255-16, 2016. Page 1, column 2: Lines 11 and 12 should read as follows. “… a frameshift that merges genes LW134a and LW134b into a gene similar to LD134 of the Neethling NI-2490 reference strain (GenBank accession no. NC_003027).”

